# CD4 Molecule Plays an Important Role in the Inflammatory Response Induced by Japanese Encephalitis Virus Infection

**DOI:** 10.3390/vetsci13030254

**Published:** 2026-03-09

**Authors:** Xinran Li, Yuanyuan Yang, Xinlei Liu, Yu Dai, Yu Gu, Ruiqin Zhang, Jiahui Li, Haodong Chen, Yi Zheng, Rui Wu

**Affiliations:** 1 Research Center for Swine Diseases, College of Veterinary Medicine, Sichuan Agricultural University, Chengdu 611130, China; 2 Sichuan Science-Observation Experiment Station of Veterinary Drugs and Veterinary Diagnostic Technology, Ministry of Agriculture, Chengdu 611330, China; 3 National Animal Experiments Teaching Demonstration Center, Sichuan Agricultural University, Chengdu 611330, China

**Keywords:** Japanese encephalitis virus, CD4, TM3 cells, STAT1, inflammatory factors

## Abstract

Japanese encephalitis virus (JEV) is an important flavivirus that causes zoonotic and arboviral diseases. Infection with JEV not only induces acute central nervous system (CNS) infectious diseases but also leads to reproductive disorders. Currently, research on the pathogenic mechanism of JEV has mainly focused on CNS inflammation caused by infection, while studies on the pathogenic mechanism of JEV targeting the reproductive system are relatively scarce. This study used TM3 cells as a model to investigate the regulatory role of the CD4 molecule in JEV infection, the STAT1 signaling pathway, and inflammatory factors. Firstly, we found that CD4 knockdown significantly inhibited JEV replication in TM3 cells. Further virus adsorption and internalization experiments confirmed that CD4 knockdown specifically impaired the early stages of JEV invasion into cells. Additionally, CD4 knockdown also drastically attenuated JEV infection-induced STAT1 phosphorylation (p-STAT1) and the production of downstream inflammatory factors. To distinguish whether CD4 affects p-STAT1 through an indirect effect of reduced viral load or its direct involvement in signal transduction, we performed experiments using RO8191, a specific agonist of the STAT1 signaling pathway. The results showed that RO8191 treatment increased the expression levels of p-STAT1 protein and inflammatory factor mRNA in both normal cells and CD4 knockdown cells, but the recovery amplitude in the CD4 knockdown group was significantly lower. In contrast, CD4 complementation significantly elevated the expression levels of p-STAT1 protein and inflammatory factor mRNA. In conclusion, this study demonstrates that the CD4 molecule positively regulates JEV proliferation in TM3 cells, while also modulating STAT1—a key factor in the STAT signaling pathway—and downstream inflammatory cytokines. Notably, this regulatory effect operates independently of viral replication. These findings provide a theoretical foundation for further elucidation of JEV pathogenic mechanisms and offer a scientific basis for the prevention and control of JEV.

## Simple Summary

TM3 cells were utilized as a model to investigate the regulatory effects of CD4 on Japanese encephalitis virus (JEV) infection, the STAT1 signaling pathway, and inflammatory factor expression. Our results demonstrate that CD4 knockdown inhibits the early adsorption, internalization, and proliferation of JEV, while concurrently downregulates phosphorylated STAT1 (p-STAT1) and inflammatory cytokines. Although treatment with the STAT agonist RO8191 failed to fully restore these signaling indicators in CD4-knockdown cells, rescuing CD4 expression significantly reversed the inhibitory effects. Studies have confirmed that, on one hand, CD4 molecules positively regulate JEV proliferation, while on the other hand, CD4 modulates STAT1 activation, and this regulation extends beyond the impact of viral replication, providing a theoretical basis for understanding the pathogenic mechanisms of JEV in the reproductive system and its prevention and control.

## 1. Introduction

Japanese encephalitis (JE) is caused by infection with Japanese encephalitis virus (JEV), a member of the Flavivirus genus within the Flaviviridae family [1]. Currently, JE is predominantly endemic in Asia, the western Pacific region, and northern Australia [2]. JEV infection can induce severe neurological inflammation, blood–brain barrier disruption, and neuronal damage [3]. Beyond the central nervous system, studies have demonstrated that JEV exhibits a distinct tropism for testicular tissues. The virus is capable of establishing persistent infections in Sertoli cells and interstitial cells of the testis, leading to reproductive disorders. For instance, JEV infection in swine herds can cause miscarriage in sows and orchitis in boars [4,5].

CD4 is conventionally recognized as a key surface marker predominantly expressed in various immune cell types, including lymphocyte subsets, monocytes, macrophages, and certain dendritic cell populations [6]. As a canonical co-receptor, CD4 strengthens the interaction between major histocompatibility complex class II (MHC II) and T cell receptors (TCRs), thereby exerting an auxiliary function in B-cell activation and the initiation of humoral immune responses. In addition to its role in antigen presentation, CD4 also participates in signal transduction events that are essential for full T-cell activation [7]. Given its pivotal role in immune regulation, aberrant expression or dysfunction of CD4 may disrupt immune homeostasis and increase host susceptibility to multiple diseases [8]. Beyond its classical immune-related functions, CD4 has been identified as an important co-factor or regulatory molecule during viral infection. It is widely involved in viral attachment, internalization, and subsequent replication within host cells. For example, during infection by RNA viruses such as human immunodeficiency virus (HIV) and simian immunodeficiency virus (SIV), CD4 acts as a primary receptor that facilitates viral binding and entry, thus improving overall infection efficiency [9]. Accumulating evidence indicates that CD4 is also expressed on certain non-immune cells. However, its specific functions in these cells, particularly in antiviral innate immunity, remain largely elusive. TM3 cells, a mouse testicular Leydig cell line, represent a well-established in vitro model for investigating the latent infection mechanisms of JEV in the reproductive system.

Activation of the innate immune response represents one of the core mechanisms by which host cells defend against viral invasion, and the interferon (IFN) signaling pathway serves a critical role in this process. Notably, almost all types of IFN exert their biological functions through the JAK-STAT signaling pathway [10,11]. Signal Transducer and Activator of Transcription 1 (STAT1) acts as a central effector molecule in the JAK-STAT cascade. Upon viral detection and subsequent IFN stimulation, STAT1 becomes phosphorylated, forms dimers, and translocates into the nucleus to drive the transcription of numerous interferon-stimulated genes (ISGs). This cascade ultimately establishes an antiviral state within host cells and orchestrates the subsequent inflammatory response [12]. Consequently, precise regulation of the JAK-STAT signaling pathway is critical for the host’s ability to clear viruses and control inflammatory damage. However, the regulation of STAT1 can be mediated not only by the classical JAK-STAT inflammatory pathway but also through JAK-independent pathways involving Syk, p38 MAPK, EGFR, and others [13,14,15]. Many viruses have evolved various strategies to suppress or evade immune responses. JEV can employ non-structural proteins such as NS1, NS4B, and NS5 to interact with different proteins in interferon-related immunosuppressive pathways, targeting the inhibition of interferon production and the antiviral effects of its signaling pathways, thereby achieving immune evasion and facilitating effective replication and persistent infection within the host [16,17,18]. The NS1’ protein of JEV is a key viral infection-enhancing factor that facilitates viral establishment and dissemination in the host by enhancing the infection of key tonsillar immune cells in pigs [19].

Currently, research on the pathogenic mechanisms of JEV primarily focuses on the central nervous system inflammation induced by its infection, while studies on the pathogenic mechanisms underlying JEV-associated reproductive system impairment remain relatively scarce. This study uses TM3 cells as an in vitro model to elucidate the role of CD4 in JEV infection and their regulatory mechanisms governing the JAK-STAT1 signaling pathway and inflammatory factors. In this study, we found that CD4 can positively regulate JEV proliferation, and our data reveal that the impact of CD4 on the STAT1 signaling pathway is intrinsic to the CD4 itself rather than an indirect effect resulting from altered viral load. This study aims to unveil the novel function of CD4 in TM3 cells, enrich the molecular regulatory mechanisms of JEV infection, and provide a new theoretical basis for a deeper understanding of JEV pathogenicity and immune evasion strategies.

## 2. Materials and Methods

### 2.1. Cells and Virus

Baby hamster kidney cells (BHK-21, ATCC, CCL-10), mouse testicular Leydig cells (TM3, ATCC, CRL-1714), human embryonic kidney 293T cells (HEK-293T, ATCC, CRL-3216), and CD4-knockdown TM3 cells (CD4.KD cells, derived from parental TM3 cells) were cultured in Dulbecco’s Modified Eagle Medium (DMEM; Gibco^®^, Thermo Fisher Scientific, Waltham, MA, USA). All media were supplemented with 10% fetal bovine serum (FBS; Hyclone, Cytiva, Marlborough, MA, USA) and 1% penicillin–streptomycin solution. Cells were maintained at 37 °C in a humidified atmosphere containing 5% CO_2_.

The Japanese encephalitis virus strain SCYA201201–1 (GenBank accession number: KU508408.1) was propagated in BHK-21 cells, and its titer was determined by plaque assay. The STAT1 agonist RO8191 (dissolved in DMSO) was purchased from MedChemExpress (MCE, Monmouth Junction, NJ, USA) and diluted to the working concentration in serum-free DMEM prior to use.

### 2.2. Plasmids and Antibodies

Plasmids psPAX (12260), pMD2.G (12259), and LentiCRISPR-V2 (52961) were obtained from Addgene (Cambridge, MA, USA). The CD4 overexpression plasmid (pEGFP-N1-CD4) was constructed and stored in our laboratory. Primer sequences used for plasmid construction, CD4 rescue experiments, and real-time quantitative reverse transcription polymerase chain reaction (RT-qPCR) are listed in Table 1. All recombinant plasmids were verified by Sanger dideoxy sequencing.

Antibodies used in this study included: anti-JEV E protein antibody (HL2517; Genetech, San Antonio, TX, USA), anti-CD4 antibody (bsm-55558R; Bioss, Beijing, China), anti-STAT1 antibody (ET1606-39), anti-p-STAT1 antibody (HA722083), anti-IL-1β antibody (HA601036; Hangzhou Huan Biotechnology Co., Ltd., Hangzhou, China), anti-β-actin antibody (AC026), horseradish peroxidase (HRP)-conjugated goat anti-rabbit IgG (AS014), and HRP-conjugated goat anti-mouse IgG (AS003; all from Abbkine Scientific Co., Ltd., Wuhan, China).

### 2.3. Western Blot Analysis

Cells were washed twice with ice-cold phosphate-buffered saline (PBS), then lysed in radioimmunoprecipitation assay (RIPA) buffer containing phenylmethylsulfonyl fluoride (PMSF; Solarbio, Beijing, China) and incubated on ice for 20 min. Lysates were centrifuged at 12,000× *g* for 5 min at 4 °C, and supernatants were collected. Protein loading buffer was added to the supernatants, followed by boiling at 100 °C for 5 min. Protein concentrations were quantified using the bicinchoninic acid (BCA) assay (Thermo Fisher Scientific), and equal amounts of total protein were loaded per lane after normalization.

Proteins were separated by sodium dodecyl sulfate–polyacrylamide gel electrophoresis (SDS-PAGE) and transferred onto polyvinylidene fluoride (PVDF) membranes (Bio-Rad Laboratories, Hercules, CA, USA). Membranes were blocked with 5% non-fat milk (Sangon Biotech, Shanghai, China; cat. no. A600669) in 0.1% Tween-20 in Tris-buffered saline (TBST) for 2 h at room temperature. Primary antibodies against CD4, STAT1, JEV E protein, and β-actin (all diluted 1:1000 in primary antibody dilution buffer; Beyotime Biotechnology, Shanghai, China) were applied and incubated overnight at 4 °C. After three 5 min washes with TBST, membranes were incubated with HRP-conjugated goat anti-rabbit/mouse IgG secondary antibodies (diluted 1:10,000) for 60 min at room temperature, followed by another three washes. Enhanced chemiluminescence (ECL) substrate (Bio-Rad) was applied according to the manufacturer’s instructions. Band intensities were quantified using ImageJ software (Version 1.53e).

### 2.4. Construction of CD4-Knockdown and CD4-Rescue TM3 Cell Lines

A CRISPR/Cas9-based gene-editing system was employed. Target-specific single-guide RNA (sgRNA) sequences against the murine *Cd4* gene were designed using the online tool CRISPR Design (https://crispr.mit.edu/, accessed on 24 August 2024): forward sequence, 5′-AAACGGTGAAGACTGTGATCTTCTTC-3′; reverse sequence, 5′-CACCGAAGAAGATCACAGTCTTCACC-3′. These oligonucleotides were cloned into the LentiCRISPR-V2 vector. Recombinant plasmids were co-transfected into HEK-293T cells together with psPAX and pMD2.G using Lipofectamine™ 3000 (Invitrogen, Carlsbad, CA, USA). After 24 h, viral supernatants were filtered and collected as lentiviral particles for transduction of exponentially growing TM3 cells.

Forty-eight hours post-infection, puromycin (final concentration: 5 μg/mL) was added for selection of transduced cells. After 7 consecutive days of selection, CD4 knockdown efficiency was confirmed by DNA sequencing and Western blot analysis. After confirmation by sequencing and protein level detection, the selected cell line was identified as a CD4 knockdown heterozygous cell line. Compared with the parental TM3 cells, the CD4 gene was significantly knocked down in this heterozygous cell line, which was subsequently used in subsequent experiments.

### 2.5. EdU Proliferation Assay

To assess cell proliferation capacity, CD4.KD and wild-type TM3 cells were seeded in 12-well plates and cultured in DMEM supplemented with 10% FBS, 100 U/mL penicillin, and 100 μg/mL streptomycin at 37 °C under 5% CO_2_. After 24 h, the BeyoClick™ Alexa Fluor 555 EdU Cell Proliferation Kit (Beyotime) was used according to the manufacturer’s protocol. Nuclei were counterstained with 4′,6-diamidino-2-phenylindole (DAPI; Beyotime) for 10 min at room temperature in the dark, and images were acquired using a fluorescence microscope. The percentage of EdU-positive cells was calculated using ImageJ software (Version 1.53e). Each experimental condition included three independent biological replicates, with one randomly selected field per well captured for analysis.

### 2.6. RNA Extraction and Quantitative Real-Time RT-qPCR

Total cellular RNA was extracted using the Trizol Total RNA Extraction Kit (Sangon Biotech, Shanghai, China). Complementary DNA (cDNA) was synthesized from the total RNA using the PrimeScript™ RT Reagent Kit (Takara Bio, Tokyo, Japan). Quantitative real-time PCR was performed using TB Green Premix Ex Taq™ II (containing Tli RNaseH Plus, Takara Bio, Tokyo, Japan) on a LightCycler 96 Real-Time PCR System (Roche, Basel, Switzerland).

The PCR amplification program was as follows: pre-denaturation at 94 °C for 30 s; followed by 40 cycles of denaturation at 94 °C for 15 s, annealing and extension at 60 °C for 30 s; finally, melting curve analysis was performed in the range of 60–95 °C. The relative expression levels of target genes were calculated using the 2^^(−ΔΔCt)^ method. GAPDH was used as the internal reference gene, and the blank control group was used as the calibration group to calculate the fold changes in gene expression.

### 2.7. Immunofluorescence Assay (IFA)

CD4.KD and TM3 cells were seeded onto glass coverslips placed in 12-well plates and infected with JEV at a multiplicity of infection (MOI) of 1. After 24 h incubation at 37 °C, cells were washed three times with PBS, fixed with 4% paraformaldehyde for 30 min, permeabilized with 0.1% Triton X-100 for 30 min at room temperature, and washed again three times with PBS. Cells were blocked with 2% bovine serum albumin (BSA) for 1 h at 37 °C. Primary anti-JEV E protein antibody (1:200 dilution) was added and incubated overnight at 4 °C. After washing with PBS containing 0.1% Tween-20 (PBST), FITC-conjugated goat anti-rabbit secondary antibody (1:500) was applied for 1 h at 37 °C, followed by three additional PBST washes. Nuclei were stained with DAPI for 10 min, and fluorescent signals were visualized using an Olympus BX63 fluorescence microscope (Tokyo, Japan).

### 2.8. Virus Adsorption and Internalization Assays

CD4.KD and wild-type TM3 cells were incubated with JEV (MOI = 10) at 4 °C for 2 h to allow viral adsorption. Unbound virions were removed by three washes with ice-cold PBS, and cells were harvested for RT-qPCR to quantify surface-bound viral RNA (adsorption).

Following adsorption, cells were shifted to 37 °C for an additional 2 h to allow internalization. Non-internalized virions were removed by three washes with ice-cold citric acid buffer (pH 3.0; Solarbio), and intracellular viral RNA was quantified by RT-qPCR (internalization).

### 2.9. Statistical Analysis

All experiments were performed with three independent replicates. Data were statistically analyzed using GraphPad Prism 8 software and expressed as mean ± standard deviation (Mean ± SD). Unpaired t-tests were used for comparisons between two groups of data, and one-way analysis of variance (one-way ANOVA) was used for comparisons among multiple groups of data. A value of *p* < 0.05 was considered statistically significant.

## 3. Results

### 3.1. Construction of CD4 Knockdown TM3 Cell Line

**Figure 1:**
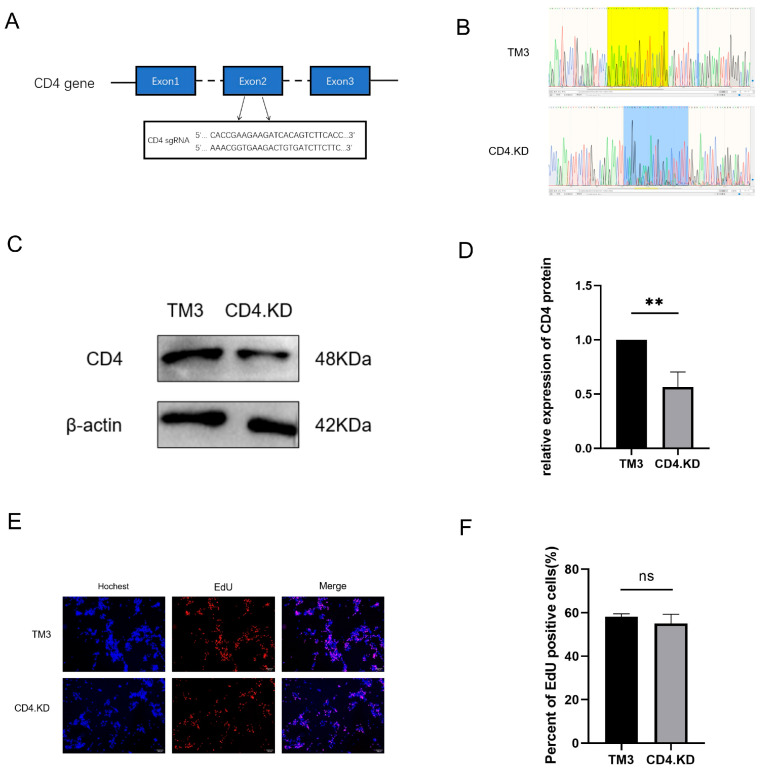
CRISPR/Cas9 gene editing technology for knockdown of the CD4 gene in TM3 cells. Note: ** indicates p<0.01, representing a statistically significant difference compared to the control group; ns indicates no significant difference. (**A**) sgRNA sequence targeting exon 2 of the CD4 gene. (**B**) DNA sequencing results for TM3 and CD4.KD cells. (**C**) Detection of CD4 protein knockdown efficiency via Western blot analysis. (**D**) Gray value analysis of the bands from three biological replicates was performed using ImageJ software to quantify the expression level of the CD4 protein. Statistical analysis results showed a significant difference in CD4 protein expression between the CD4.KD cells and the TM3 cells (*p* = 0.0054, *p* < 0.05). (**E**) Fluorescence microscopy results following EdU treatment in TM3 and CD4.KD cells. (**F**) At least three images were taken for each sample to calculate the percentage of EdU-positive cells, with the mean reported as the final data (the original pictures can be found in Figure S1).

A single-guide RNA (sgRNA) targeting the second exon of the CD4 gene was designed using an online CRISPR design tool (Figure 1A). The annealed sgRNA duplex was cloned into the linearized LentiCRISPR-V2 plasmid, which had been predigested with the restriction enzyme BsmB I. The TM3 cell line with stable CD4 gene knockdown, generated via the CRISPR/Cas9 gene-editing system, was designated as CD4.KD cells. Genomic DNA was extracted from both wild-type TM3 and CD4.KD cells for sequencing validation (Figure 1B). Quantitative real-time PCR (qPCR) and Western blot analyses further confirmed efficient knockdown of CD4 protein expression in the engineered cells (Figure 1C–E). Moreover, results from the EdU cell proliferation assay demonstrated that CD4 knockdown exerted no significant effect on the proliferative capacity of TM3 cells (Figure 1F).

### 3.2. CD4 Gene Knockdown Inhibits JEV Proliferation

**Figure 2:**
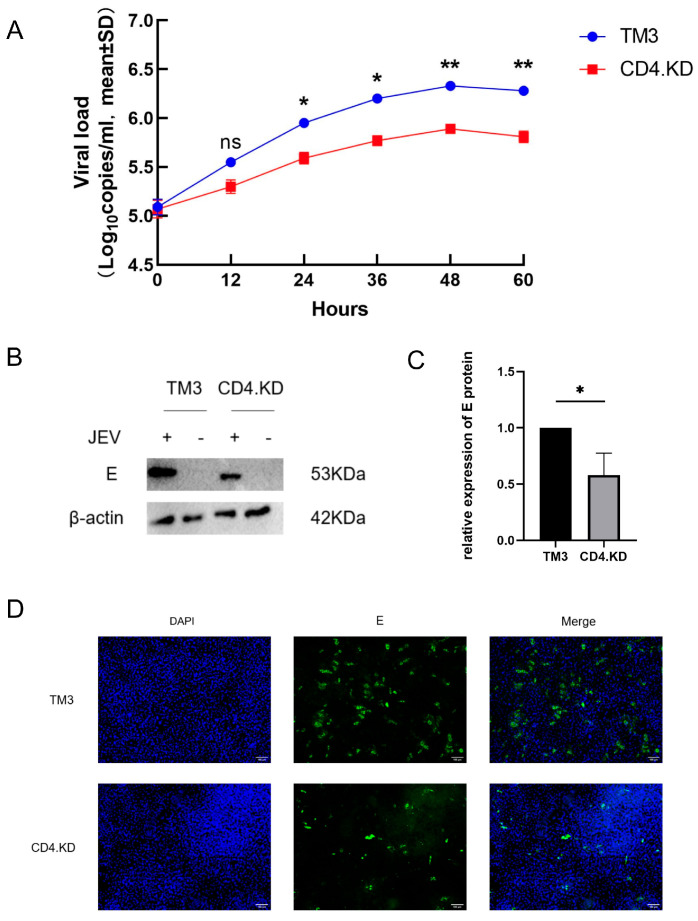
CD4 Gene Knockdown Inhibits Proliferation of Japanese Encephalitis Virus in TM3 Cells. Note: * indicates p<0.05, ** indicates p<0.01, ns: not significant, representing a statistically significant difference compared to the control group. (**A**) Samples of TM3 and CD4.KD cells infected at specified time points were collected for relative quantitative RT-qPCR analysis of E gene expression, and growth curves were plotted. (**B**) TM3 and CD4.KD cells were infected with JEV (MOI = 1) for 48 h, and cell samples were collected for Western blot analysis of E protein. (**C**) To quantify the expression level of the E protein, gray value analysis of the bands from three biological replicates was performed using ImageJ software. Statistical analysis revealed a significant difference in E protein expression between the CD4.KD cells and the TM3 cells (*p* = 0.0204, *p* < 0.05). (**D**) Fluorescence staining was conducted to assess E protein expression in TM3 and CD4.KD cells infected with JEV (MOI = 1) for 48 h (the original pictures can be found in Figure S2).

To determine the impact of CD4 gene knockdown on JEV proliferation in TM3 cells, virus growth curves were plotted for CD4.KD and TM3 cells infected with JEV (MOI = 1), with samples collected every 12 h. The growth curves revealed that CD4 knockdown significantly inhibited viral release (Figure 2A). Based on the growth curves, CD4.KD and TM3 cells were collected 48 h post-infection with JEV (MOI = 1) and examined for E protein expression using Western blot analysis. The expression level of E protein was found to be significantly lower in CD4.KD cells compared to TM3 cells (Figure 2B,C). This finding was further validated using indirect immunofluorescence, where rabbit anti-E antibody was used as the primary antibody, and FITC-conjugated goat anti-rabbit antibody was used as the secondary antibody to stain the E protein, with Hoechst staining for the cell nuclei (Figure 2D). The results demonstrated that the viral load in CD4 knockdown cells was significantly lower than in normal cells, indicating that CD4 gene knockdown can inhibit JEV proliferation.

### 3.3. CD4 Overexpression Promotes JEV Infection

**Figure 3:**
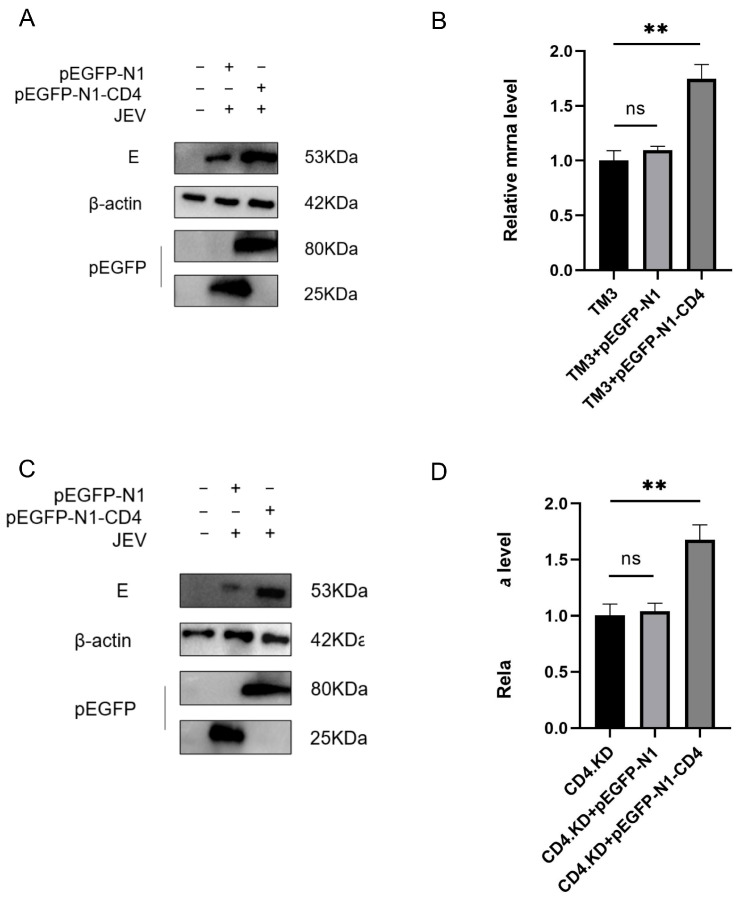
CD4 Protein Plays a Positive Regulatory Role in Japanese Encephalitis Virus Proliferation. pEGFP-N1 and pEGFP-N1-CD4 plasmids were transfected into TM3 or CD4.KD cells. Note: ** indicates p<0.01, ns: not significant, representing a statistically significant difference compared to the control group (**A**) TM3 cells were infected with JEV (MOI = 1) for 48 h. (**B**) The expression levels of E mRNA in each group of cells were detected by RT-qPCR. Statistical analysis revealed a highly significant difference between the TM3 group and the TM3 + pEGFP-N1-CD4 group (*p* = 0.0012, *p* < 0.01). (**C**) CD4.KD cells were infected with JEV (MOI = 1) for 48 h, followed by protein blot analysis of viral load. (**D**) The expression levels of E mRNA in each group of cells were detected by RT-qPCR. Statistical analysis revealed a highly significant difference between the CD4.KD group and the CD4.KD + pEGFP-N1-CD4 group (*p* = 0.0022, *p* < 0.01) (the original pictures can be found in Figure S3).

pEGFP-N1 and pEGFP-N1-CD4 were transfected into CD4.KD and TM3 cells, followed by JEV infection for 48 h (MOI = 1). The changes in E gene mRNA levels and E protein expression levels were measured using qPCR and Western blot analysis. The expression levels of E protein in both the replenishment and overexpression groups were significantly elevated (Figure 3A,C). The qPCR results were consistent with the Western blot findings, showing a significant upregulation of mRNA levels in the replenishment and overexpression groups, while there was no statistically significant difference between the empty plasmid group and normal cells (Figure 3B,D). These results suggest that CD4 has a positive regulatory role in JEV proliferation.

### 3.4. CD4 Gene Knockdown Inhibits JEV Proliferation During the Early Stage of Infection

**Figure 4:**
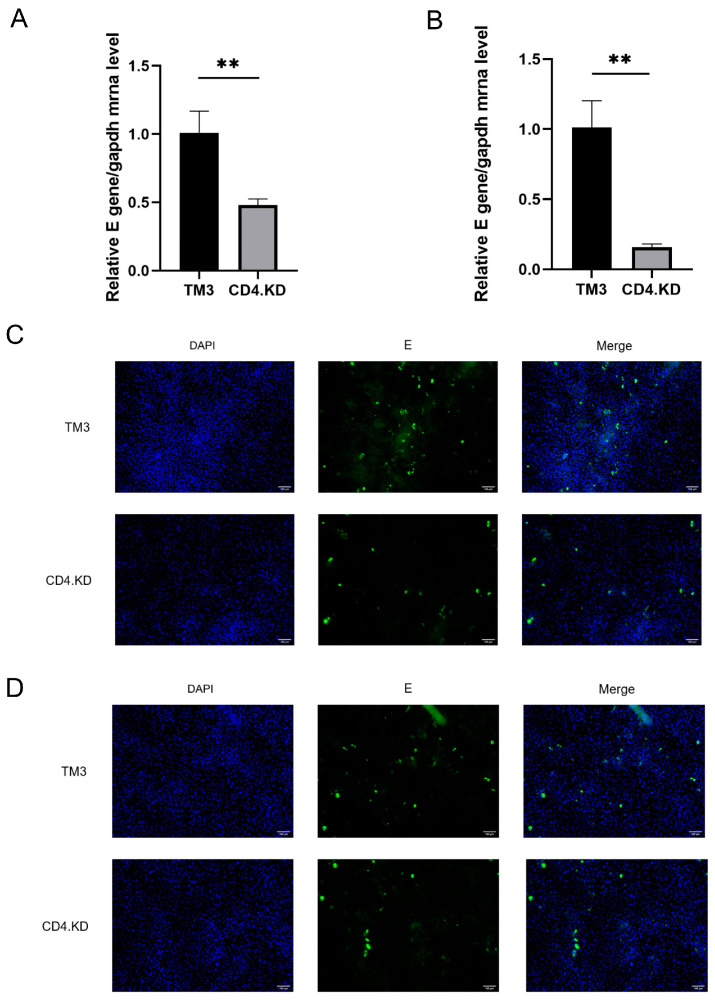
CD4 Knockdown Negatively Affects the Early Stages of JEV Invasion in TM3 Cells (Adsorption and Internalization). Note: ** indicates p<0.01, representing a statistically significant difference compared to the control group (**A**) TM3 and CD4.KD cells were infected with JEV at an MOI of 10 and incubated at 4 °C for 2 h. The levels of cell surface-bound JEV E mRNA were quantified by RT-qPCR to assess viral adsorption. Statistical analysis revealed a significant difference in viral adsorption between the two groups (*p* = 0.0052, *p* < 0.01). (**B**) TM3 and CD4.KD cells were infected with JEV at an MOI of 10 and incubated at 4 °C for 2 h. Unadsorbed virus was removed, and pre-chilled DMEM containing 2% FBS was added. The cells were then rapidly transferred to a 37 °C, 5% CO_2_ incubator and incubated for an additional 2 h. The levels of cell-associated JEV E mRNA were quantified by RT-qPCR to assess viral internalization. Statistical analysis revealed a significant difference in viral internalization between the two groups (*p* = 0.0016, *p* < 0.01). (**C**) Fluorescence staining was performed to detect the expression of E protein, reflecting the amount of virus adsorption. (**D**) Fluorescence staining was performed to detect the expression of E protein, reflecting the amount of virus internalization (the original pictures can be found in Figure S1).

CD4.KD and TM3 cells were infected with JEV at an MOI of 10, and the transcription levels of the JEV E gene were measured to indirectly assess the amount of viral particle adsorption and internalization. The experimental results indicated a significant difference in the transcription levels of the JEV E gene during the virus adsorption and internalization stages between CD4.KD and TM3 cells (Figure 4A,B). This was further validated using indirect immunofluorescence, where rabbit anti-E antibody was used as the primary antibody and FITC-conjugated goat anti-rabbit antibody was used to stain the E protein, along with Hoechst staining for the cell nuclei (Figure 4C,D). The results showed that the amount of virus adsorption on CD4 knockdown cells was significantly lower than that in normal cells, suggesting that CD4 protein plays a promoting role in the invasion process of JEV.

### 3.5. CD4 Gene Knockdown Reduces p-STAT1 Protein Levels in TM3 Cells Post-JEV Infection

**Figure 5:**
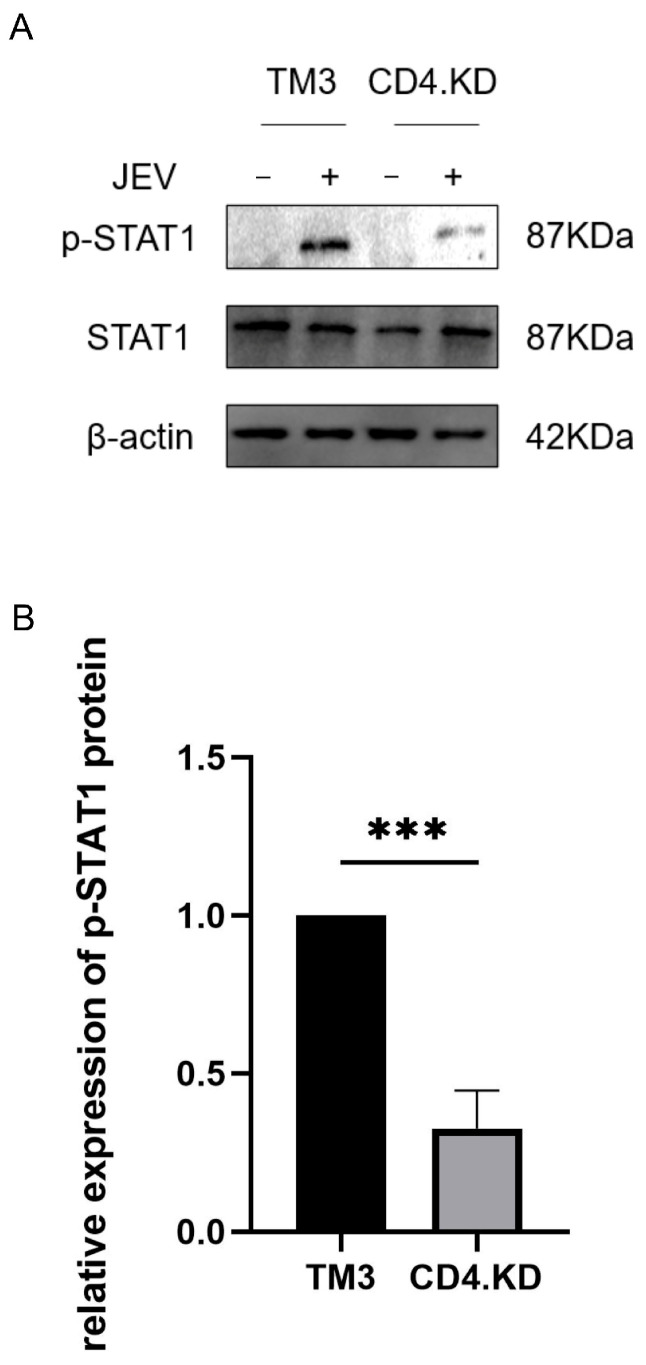
CD4 Knockdown Reduces p-STAT1 Protein Levels in TM3 Cells Post-JEV Infection. Note: *** indicates p<0.001, representing a statistically significant difference compared to the control group. (**A**) TM3 and CD4.KD cells were infected with JEV (MOI = 1) for 48 h, and cell samples were collected for Western blot analysis to assess p-STAT1 protein levels. (**B**) To quantify the expression level of the p-STAT1 protein, gray value analysis of the bands from three biological replicates was performed using ImageJ software. Statistical analysis revealed a significant difference in p-STAT1 protein expression between CD4.KD and TM3 cells (*p* = 0.0006, *p* < 0.01) (the original pictures can be found in Figure S5).

CD4.KD and TM3 cells were infected with JEV at an MOI of 1 and analyzed 48 h later using Western blot to detect p-STAT1 protein levels in both groups. The results showed that the expression of p-STAT1 protein in the CD4 knockdown group was significantly lower than that in the control group, suggesting that CD4 knockdown may affect the changes in the key factor p-STAT1 in the JAK-STAT1 signaling pathway triggered by JEV infection (Figure 5).

### 3.6. CD4 Gene Replenishment Reverses the Inhibitory Effect of CD4 Knockdown on the Activation of the JAK-STAT1 Inflammatory Pathway Agonist RO8191

**Figure 6:**
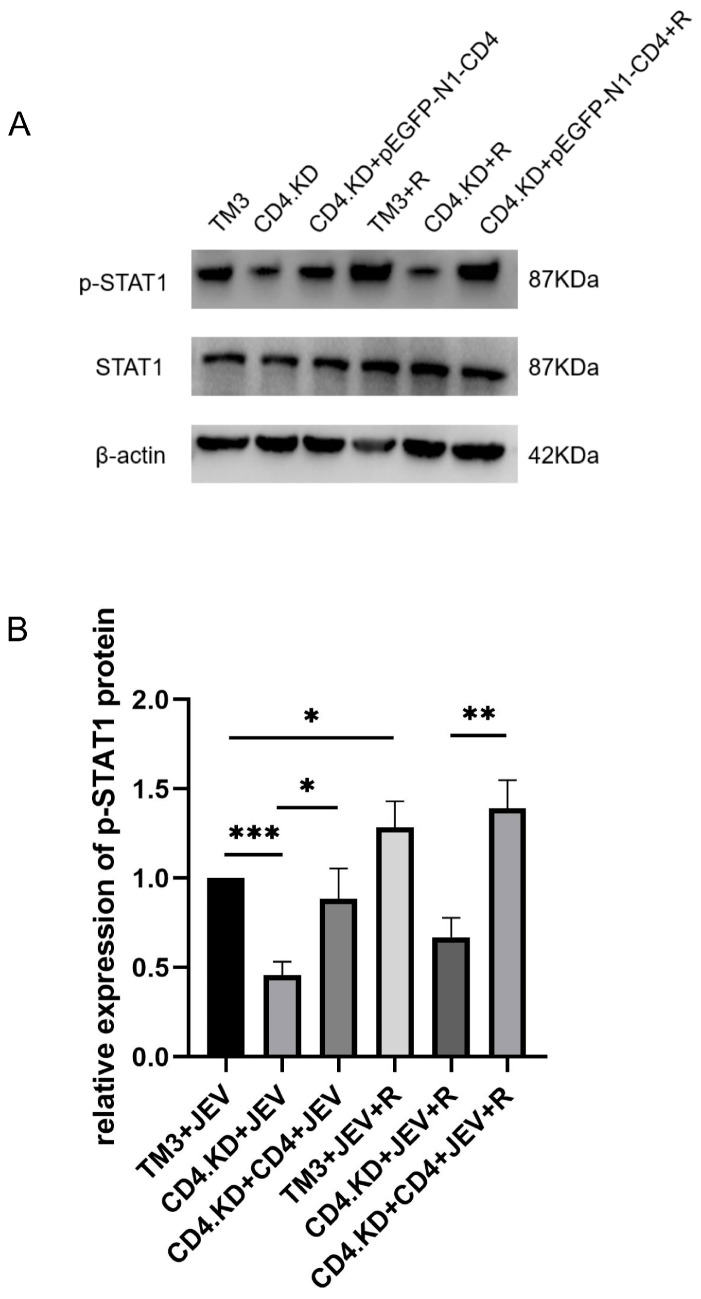
CD4 Gene Knockdown Significantly Inhibits the Activating Effect of RO8191 on the JAK-STAT1 inflammatory pathway, and This Inhibitory Phenotype Can Be Reversed by CD4 Replenishment. Note: * indicates *p* < 0.05, ** indicates *p* < 0.01, and *** indicates *p* < 0.0001, all representing significant differences compared with the control group. (**A**) Three models were established: normal TM3 cells, CD4.KD cells, and CD4 replenished cells. Each group was divided into a control group and an RO8191 pre-treatment experimental group. After inoculating with JEV at an MOI of 1 and culturing for 48 h, Western blot analysis was performed to detect the expression levels of p-STAT1 protein. (**B**) Gray value analysis of the bands from three biological replicates was performed using ImageJ to quantify the p-STAT1 protein (the original pictures can be found in Figure S6).

Since CD4 knockdown inhibits the adsorption and internalization of JEV in TM3 cells, this study aimed to clarify whether the regulatory role of CD4 in the JEV-induced JAK-STAT1 signaling pathway originates from the CD4 molecule itself or is an indirect effect due to changes in viral load. Three cell models were established: TM3 cells, CD4.KD cells, and CD4 replenished cells. The experimental group was treated in advance with the JAK-STAT1 inflammatory pathway agonist RO8191, while the control group received no treatment. Cell samples were collected 48 h after JEV infection (MOI = 1), and Western blot analysis was used to detect the expression levels of p-STAT1 and total STAT1 proteins in each group. The results showed that in normal TM3 cells, the p-STAT1 level in the RO8191 treated group significantly increased compared to the untreated infected group, clearly indicating the agonist’s effect on activating the JAK-STAT1 signaling pathway. In CD4.KD cells, the p-STAT1 level in the RO8191 treated group showed only a slight increase compared to the untreated infected group and was significantly lower than in the normal cell treatment group. In CD4 replenished cells, the p-STAT1 level in the RO8191 treated group significantly increased compared to the untreated infected group (Figure 6). These results indicate that CD4 gene knockdown significantly inhibits the activating effect of RO8191 on the JAK-STAT1 signaling pathway, and this inhibitory phenotype can be reversed by CD4 replenishment. This suggests that CD4 modulates STAT1 activation, and this regulatory effect extends beyond the impact of viral replication.

### 3.7. CD4 Gene Replenishment Reverses the Inhibitory Effect of CD4 Knockdown on the Production of Downstream Inflammatory Factors in the JAK-STAT1 Inflammatory Pathway

**Figure 7:**
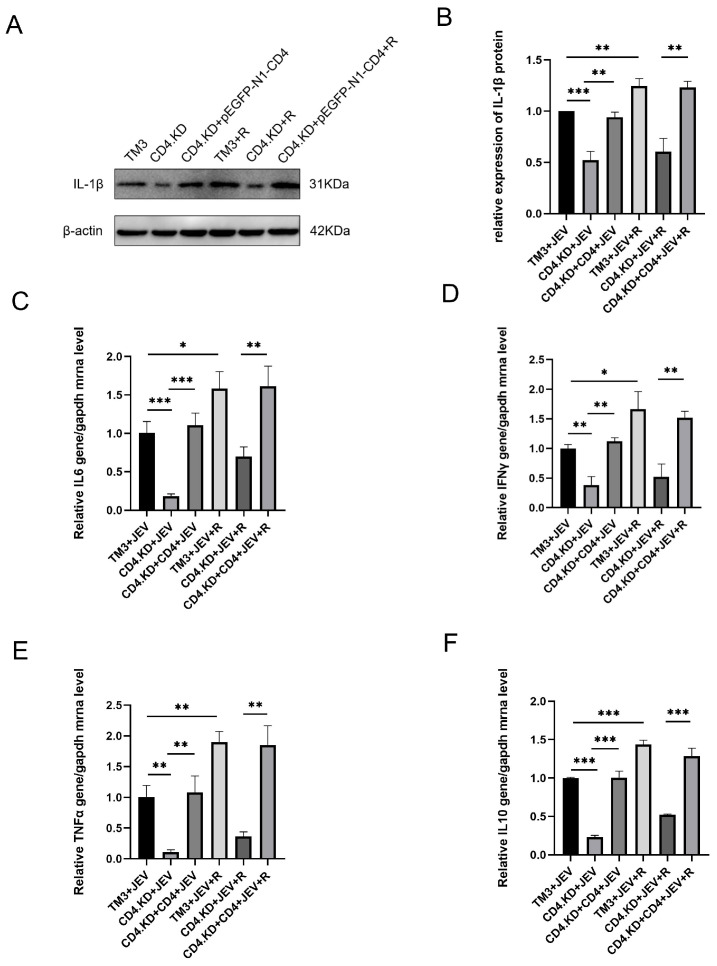
CD4 Replenishment Reverses the Inhibitory Effect of CD4 Knockdown on the Production of Inflammatory Factors (IL-1β, IL-6, IFN-γ, TNF-α, IL-10) Induced by JEV Infection in TM3 Cells. Note: * indicates *p* < 0.05, ** indicates *p* < 0.01, and *** indicates *p* < 0.0001, all representing significant differences compared with the control group. Three models were established: normal TM3 cells, CD4.KD cells, and CD4 replenished cells. Each group was divided into a control group and an RO8191 pre-treatment experimental group. After inoculating with JEV at an MOI of 1 and culturing for 48 h: (**A**) Western blot analysis was performed to detect the expression levels of IL-1β protein. (**B**) Gray value analysis of the bands from three biological replicates was performed using ImageJ to quantify the IL-1β protein. (**C**) The expression levels of IL6 mRNA in each group of cells were detected by RT-qPCR. (**D**) The expression levels of IFN-γ mRNA in each group of cells were detected by RT-qPCR. (**E**) The expression levels of TNF-α mRNA in each group of cells were detected by RT-qPCR. (**F**) The expression levels of IL-10 mRNA in each group of cells were detected by RT-qPCR (the original pictures can be found in Figure S7).

Using the same three cell models as in Section 3.6, the levels of IL-1β, TNF-α, IL-6, and IFN-γ in these cells of each cell group were collected and measured. The results showed that in the normal control group, the RO8191 treatment group exhibited significantly increased levels of all three inflammatory factors compared to the untreated infected group. In the CD4 knockdown group, the RO8191 treatment group showed only a slight increase in inflammatory factor levels, which was significantly lower than the levels in the normal control group after treatment. In the CD4 replenished group, the RO8191 treatment group displayed significantly increased levels of inflammatory factors compared to the untreated infected group (Figure 7). These findings indicate that the CD4 molecule influences JEV infection-induced production of downstream inflammatory cytokines by affecting STAT1 phosphorylation and activation.

## 4. Discussion

Previous studies have found that the CD4 gene is involved in the lifecycle of JEV, and there exists a co-localization phenomenon between CD4 protein and E protein within the cells, suggesting that the interaction between CD4 and E protein may play an important role in JEV infection and the virus lifecycle [20]. Currently, our research on the CD4 gene primarily focuses on T cell activation, regulation of immune response, and the association processes between innate and adaptive immunity [21]. As a classic transmembrane glycoprotein, CD4 has traditionally been regarded as a marker of immune cells. However, recent studies have confirmed that CD4 is functionally expressed in a variety of non-immune cells. These studies demonstrate that CD4 is not exclusive to immune cells and can exert non-classical functions in non-immune cell populations, which also provides theoretical support for the regulatory role of CD4 in TM3 cells [22,23,24,25]; however, data on the cellular surface localization of the CD4 molecule are still lacking. The core functions of the CD4 molecule are not limited to immune cell activation and immune response regulation [6], growing evidence in recent years has revealed that it plays a pivotal role in the infection processes of various viruses. For example, CD4 serves as the primary receptor that mediates HIV-1 entry into host cells. The binding of CD4 to HIV-1 gp120 induces conformational rearrangements in the viral glycoprotein, which exposes specific chemokine receptor-binding sites. Subsequent interactions between chemokine receptors and the gp120CD4 complex trigger further structural changes, ultimately promoting fusion between the viral envelope and the host cell membrane [26]. HIV1 is recognized as the prototypical pathogen of acquired immunodeficiency syndrome (AIDS), whereas HIV2 represents a second genetically distinct human lentivirus. Both HIV1 and HIV2 exhibit close genetic homology to simian immunodeficiency viruses (SIV) [27]. Nucleotide sequence similarity between HIV1 and SIV is approximately 40%, while that between HIV2 and SIV reaches around 75% [28]. All three viral lineages recognize the same CD4 molecule as their cellular receptor, indicating that CD4 acts as a conserved entry receptor for HIV1, HIV2, and SIV [9]. Currently, some progress has been made in understanding the interactions between CD4 and other viruses. However, research on the interaction between CD4 and JEV is still relatively scarce, particularly concerning the specific regulatory mechanisms of CD4 on host cells after JEV infection and its role in the immune response induced by JEV infection, with few related reports available, indicating a significant research gap.

Currently, research on the pathogenic mechanisms of JEV primarily focuses on the central nervous system inflammation it induces, while studies regarding its pathogenic mechanisms in the reproductive system are relatively limited. Testicular interstitial cells, being the primary site for the synthesis and secretion of testosterone in male animals, play a crucial role, as their functional integrity and cellular homeostasis directly determine the health of the male reproductive system [29]. The damage and inflammatory responses in testicular interstitial cells induced by JEV infection are likely to be key factors contributing to reproductive disorders in boars. TM3 cells, as a model for testicular interstitial cells, can effectively simulate the physiological state of reproductive system cells and are therefore an ideal cell model for studying JEV infection in the reproductive system and inducing inflammatory responses. To explore the function and mechanism of CD4 in JEV infection, this study established an in vitro cell infection model using TM3 cells. It has been successfully validated that JEV can infect TM3 cells in vitro and continue to induce cytopathic effects (CPEs) stably even after multiple passages. Techniques such as RT-PCR and indirect immunofluorescence have confirmed the presence of JEV NS1 protein and its nucleic acid sequences in cells after several passages, demonstrating that JEV can replicate and amplify in TM3 cells, providing a stable and reliable in vitro model for subsequent experiments. Moreover, as testicular interstitial cells, TM3 cells align well with the objectives of this study and can be utilized to investigate the pathogenic mechanisms of JEV infection-induced testicular inflammation, thereby establishing a critical link for the investigation of reproductive system lesions in vivo. In this experiment, a CD4 gene knockdown TM3 cell line was constructed using CRISPR/Cas9 and designated as CD4.KD. Western blot and indirect immunofluorescence results indicated that CD4 knockdown negatively affected JEV proliferation. Additionally, overexpression of the CD4 gene in TM3 cells, as well as the restoration of the CD4 gene in CD4.KD cells, confirmed that CD4 plays a positive regulatory role in JEV proliferation.

To further elucidate the specific mechanisms by which CD4 knockdown inhibits JEV proliferation, this study conducted adsorption and internalization experiments. The JEV infection in this experiment was performed at an MOI of 10 to ensure sufficient viral binding to TM3 and CD4.KD cells, compensating for the low binding efficiency associated with the short incubation period. This allowed for the stable detection of viral adsorption and internalization signals and enabled accurate distinction between the two cell groups. RT-qPCR and IFA results showed that CD4 knockdown significantly reduces cell-associated viral RNA levels during the early stage of infection; however, due to limitations of the experimental data, a definitive conclusion cannot be drawn regarding the decisive effect of CD4 knockdown on JEV attachment and internalization.

The STAT protein family comprises seven members, namely STAT1, STAT2, STAT3, STAT4, STAT5a, STAT5b, and STAT6. Each STAT isoform contains multiple conserved functional domains, including an N-terminal domain, a coiled-coil domain, a DNA-binding domain, a linker domain, an SRC homology 3 (SH3) domain, an SRC homology 2 (SH2) domain, and a C-terminal transactivation domain. Among these regions, the SH2 domain is primarily responsible for determining the specific binding interactions between STAT proteins and their corresponding receptors [30]. STAT proteins are canonical substrates of JAKs. The JAK-STAT signaling pathway was initially discovered in investigations of interferon-mediated transcriptional activation [31]. JAKs represent a family of intracellular non-receptor tyrosine kinases, consisting of four members: JAK1, JAK2, JAK3, and tyrosine kinase 2 (TYK2). These kinases mediate phosphorylation events on target tyrosine residues via either auto- or trans-phosphorylation mechanisms [32]. This pathway is sequentially composed of tyrosine kinase-associated receptors, Janus kinases (JAKs), and signal transducers and activators of transcription (STATs) [33]. Upon the binding of cytokines such as interleukins, interferons, and growth factors to their respective cell-surface receptors, JAKs become activated and undergo auto-phosphorylation. Activated JAKs then recruit and phosphorylate cytoplasmic STAT proteins, which subsequently dimerize and translocate into the nucleus. Within the nucleus, the STAT dimers bind to specific promoter sequences and initiate the transcription of downstream target genes involved in inflammatory and immune responses [34,35]. The JAK-STAT cascade functions as a critical inflammatory signaling axis during innate immune defense, particularly in the context of viral infection. Type I interferons (IFN-I) are potent inducers of STAT1 phosphorylation, and phosphorylated STAT1 (p-STAT1) represents its functionally active form. Activated p-STAT1 modulates the expression of multiple downstream inflammatory mediators, including IL-1β and TNF-α, and promotes the expression of interferon-stimulated genes (ISGs). This series of events initiates the host antiviral immune program and restricts viral replication, positioning the JAK-STAT pathway as a vital signaling hub connecting viral sensing to inflammatory reactions [36]. The JAK-STAT pathway is also closely associated with infections caused by members of the Flaviviridae family. Mounting evidence indicates that flaviviruses such as West Nile virus and dengue virus can either activate or antagonize the JAK-STAT cascade, thereby regulating viral replication and host immune responses. These observations suggest that the JAK-STAT pathway may serve as an evolutionarily conserved regulatory target during flavivirus infection, and further exploration of this pathway will help clarify the pathogenic mechanisms of flaviviruses [16]. Previous studies have demonstrated that abnormal activation of the JAK-STAT signaling pathway can trigger excessive inflammatory factor release in testicular tissues [37,38], leading to interstitial cell damage and reproductive dysfunction. The activation of STAT1 is not solely dependent on the JAK kinase pathway; various upstream molecules closely associated with inflammatory responses can also participate in this process. For instance, epidermal growth factor receptor (EGFR) acts as a direct upstream kinase of STAT1 and can directly phosphorylate STAT1 at Tyr701 in a JAK-independent manner, thereby inducing its activation [13]. In addition, p38 MAPK serves as a key upstream kinase that mediates STAT1 activation in response to interferon stimulation. p38 MAPK directly catalyzes the phosphorylation of STAT1 at Ser727 and regulates the downstream cPLA2ISGF3 signaling module, thereby enhancing the transcriptional activity of STAT1. This mechanism ensures efficient interferon-induced, STAT1-dependent gene expression and antiviral activity, representing an important auxiliary pathway for STAT1 activation beyond the classical JAKSTAT cascade [14]. STAT1 can also be activated by spleen tyrosine kinase (Syk) downstream of the retinoic acid-inducible gene I/mitochondrial antiviral signaling protein (RIGI/MAVS) axis. Studies have shown that Syk deficiency impairs the tyrosine phosphorylation of STAT1, which is critical for mounting an effective early antiviral immune response [15].

It should be noted that all experiments involved in this study were performed in a Class P2+ biosafety laboratory, which has obtained official institutional biosafety certification. The operation of the P2+ biosafety laboratory strictly complies with national and institutional biosafety management regulations, ensuring the safety of all experimental processes, effectively mitigating biosafety risks, and providing a safety guarantee for the reliability of the experimental results.

This study found that CD4 knockdown led to a significant decrease in the expression of p-STAT1 protein following JEV infection in TM3 cells, while the production of downstream inflammatory factors was also inhibited. However, a key question arose: Does the regulatory effect of the CD4 molecule on the STAT1 inflammatory pathway arise from the intrinsic regulatory function of CD4 itself, or is it an indirect consequence of CD4 knockdown suppressing JEV entry, thereby reducing intracellular viral load and subsequently attenuating virus-induced stimulation of the STAT1 pathway, ultimately leading to decreased p-STAT1 expression and reduced production of inflammatory cytokines?

To clarify this question, this study conducted additional CD4 gene rescue experiments, setting up three model groups: normal TM3 cells, CD4.KD cells, and CD4 knockdown rescue cells. Each group was further divided into a control group and a pre-treatment group with the STAT1 signaling pathway-specific agonist RO8191. All cells were infected with JEV at an MOI of 1 to mimic the physiological conditions of natural JEV infection and to avoid non-specific cellular damage caused by a high MOI, cells were collected after 48 h of culture to detect the protein expression levels of p-STAT1 and total STAT1, as well as the levels of four inflammatory cytokines: IL-1β, TNF-α, IL-6, and IFN-γ.

The experimental results showed that in normal TM3 cells, the p-STAT1 protein level in the RO8191 pre-treatment group significantly increased compared to the untreated infection group, confirming the clear activation effect of RO8191 on the STAT1 signaling pathway. Correspondingly, the levels of the four inflammatory factors in these cells also significantly increased compared to the untreated infection group, indicating that activation of the STAT1 signaling pathway effectively promotes the production of downstream inflammatory factors. In CD4.KD cells, the p-STAT1 level in the RO8191 pre-treatment group showed only a slight increase compared to the untreated infection group, and the results of the inflammatory factor assays exhibited a similar trend, suggesting that CD4 knockdown suppresses the activation effect of RO8191 on the STAT1 signaling pathway, thereby inhibiting the production of downstream inflammatory factors.

In the CD4 rescue cells, the p-STAT1 level in the RO8191 pre-treatment group significantly increased compared to the untreated infection group, and the levels of the four inflammatory factors in these cells also significantly increased, consistent with the changes in p-STAT1 expression, indicating that CD4 gene rescue can effectively reverse the inhibitory effect of CD4 knockdown on the activation of the STAT1 signaling pathway and the production of downstream inflammatory factors.

The above experimental results demonstrate that CD4 knockdown significantly inhibits the activating effect of RO8191 on the STAT1 pathway, and this inhibitory phenotype can be reversed by CD4 complementation. This phenomenon suggests that the CD4 molecule itself plays an important role in modulating the STAT signaling pathway. Combined with previous experimental findings, CD4 not only positively regulates viral proliferation in TM3 cells by participating in the early invasion process of JEV but also influences the phosphorylation and activation of STAT1 and the production of downstream inflammatory cytokines. This dual role of the CD4 molecule highlights its important position in JEV infection of reproductive system cells and the induction of inflammatory responses.

Using TM3 cells as a model, this study systematically investigated the influence of the CD4 molecule on JEV infection and the STAT1 inflammatory pathway. The results clarify that CD4 positively regulates viral proliferation by participating in the early invasion of JEV; meanwhile, CD4 is involved in modulating the activation of the STAT1 pathway and the production of downstream inflammatory cytokines, and this modulatory effect is independent of its impact on viral load. These findings provide a new theoretical perspective for understanding the pathogenic mechanisms of JEV in the reproductive system and offer a novel potential target and scientific basis for the prevention and control of JEV-associated diseases.

STAT1 is the core regulatory molecule investigated in this study for its role in the inflammatory response induced by JEV infection in Leydig cells. Although this study confirms that CD4 regulates the phosphorylation activation of STAT1 and the production of downstream inflammatory factors, whether this regulation is mediated through classical JAK-STAT inflammatory pathways or via JAK-independent pathways, and the specific upstream molecules and signal transduction mechanisms involved, requires further in-depth analysis and validation in subsequent experiments.

As a transmembrane glycoprotein, CD4 possesses two critical, highly conserved signaling motifs within its cytoplasmic tail—motifs that are pivotal for its signal transduction capabilities and form the basis for its modulation of downstream signaling cascades. Specifically, a conserved CxCP motif (corresponding to C420/C422 in mice) is present in the CD4 cytoplasmic tail, which facilitates a zinc-dependent association with Lck, a member of the Src-family kinases. Within canonical T-cell signaling cascades, this CxCP-Lck interaction triggers TCR signal initiation through the phosphorylation of immunoreceptor tyrosine-based activation motifs (ITAMs) located in the CD3 complex [39]. Furthermore, the CD4 cytoplasmic tail contains a membrane-proximal CVRC motif (C394/C397 in humans) that undergoes palmitoylation, a post-translational modification that tethers CD4 to lipid rafts and boosts its signal transduction efficiency without relying on Lck binding [40]. While the transmembrane domain of CD4 also includes a GGxxG motif that aids in Lck-independent signaling, this particular motif does not mediate CD4 dimerization nor does it serve as the core element responsible for regulating STAT1-related signaling pathways [41].

Based on the structural characteristics of the CD4 cytoplasmic domain and the experimental results of this study, we speculate that the CD4 cytoplasmic tail may regulate STAT1-related signaling pathways in TM3 cells through the following potential mechanisms: First, the CxCP motif in the CD4 cytoplasmic tail, known to bind Lck, may interact with other Src-family kinases in TM3 cells, thereby initiating a downstream kinase cascade, promoting the phosphorylation and nuclear translocation of STAT1, and ultimately regulating the activation state of the STAT1 pathway. Second, the palmitoylation modification of the CVRC motif in the CD4 cytoplasmic tail can anchor CD4 to lipid raft microdomains. This membrane localization may facilitate the colocalization of CD4 with STAT1 or its upstream regulatory molecules in TM3 cells, improving signal transduction efficiency and consequently influencing the activation of the STAT1 pathway. Third, the CxCP and CVRC motifs may act synergistically: the CxCP motif mediates the recruitment and activation of upstream kinases, while the CVRC motif optimizes the membrane localization of CD4 to ensure efficient signal transmission. Together, they participate in the regulatory role of CD4 on the STAT1 pathway during JEV infection and inflammatory responses in TM3 cells.

The above speculation is hypothetical, and the underlying mechanism requires further investigation.

## 5. Conclusions

CD4 knockdown significantly inhibits the early invasion process of JEV in TM3 cells, leading to a marked reduction in intracellular viral load and positively regulating viral proliferation in reproductive system cells. Furthermore, CD4 knockdown substantially downregulates the expression level of p-STAT1 protein in JEV-infected TM3 cells while attenuating the production of downstream inflammatory cytokines, including IL-1β, TNF-α, IL-6, and IFN-γ. Importantly, the effect of CD4 on the STAT1 pathway is intrinsic to the CD4 molecule itself rather than an indirect consequence of reduced viral load. This dual role of the CD4 molecule—both regulating JEV proliferation through involvement in viral invasion and influencing STAT1 pathway activation and subsequent inflammatory cytokine production—highlights its critical importance in JEV infection-induced inflammatory responses in Leydig cells.

## Figures and Tables

**Table 1:** Primers used for plasmid construction and RT-qPCR in this study.

Primer Name	Forward (5′→ 3′)	Reverse (5′→ 3′)
Q-E	CAGTGGAGCCACTTGGGTG	TTGTGAGCTTCTCCTGTCG
Q-IFN-γ	GGAGGAACTGGCAAAAGGATG	GTTGCTGATGGCCTGATTGT
Q-IL-6	GGGACTGATGCTGGTGACAA	ACAGGTCTGTTGGGAGTGGT
Q-IL-1β	TGCCACCTTTTGACAGTGATG	TGATGTGCTGCTGCGAGATT
Q-IL-10	GGTTGCCAAGCCTTATCGGA	ACCTGCTCCACTGCCTTGCT
Q-TNF-α	TAGCCCACGTCGTAGCAAAC	TGTCTTTGAGATCCATGCCGT
Q-GAPDH	CATCACTGCCACCCAGAAGAC	ATTGGGGGTAGGAACACGGA

## Data Availability

The original contributions presented in this study are included in the article/Supplementary Material. Further inquiries can be directed to the corresponding author.

## Supplementary Materials

The following supporting information can be downloaded at: https://www.mdpi.com/article/10.3390/vetsci13030254/s1, Figure S1: Original images of Figure 1; Figure S2: Original images of Figure 2; Figure S3: Original images of Figure 3; Figure S4: Original images of Figure 4; Figure S5: Original images of Figure 5; Figure S6: Original images of Figure 6; Figure S7: Original images of Figure 7.
