# Supplementary material for: CD4 Molecule Plays an Important Role in the Inflammatory Response Induced by Japanese Encephalitis Virus Infection
Source: Vet Sci. 2026 Mar 9;13(3):254. doi: 10.3390/vetsci13030254 (PMC13030670; doi:10.3390/vetsci13030254)
Supplement: Supplementary file 1 [file vetsci-13-00254-s001.zip › vetsci-4166193-supplementary/figure S5.pdf]

FIGURE 5 A

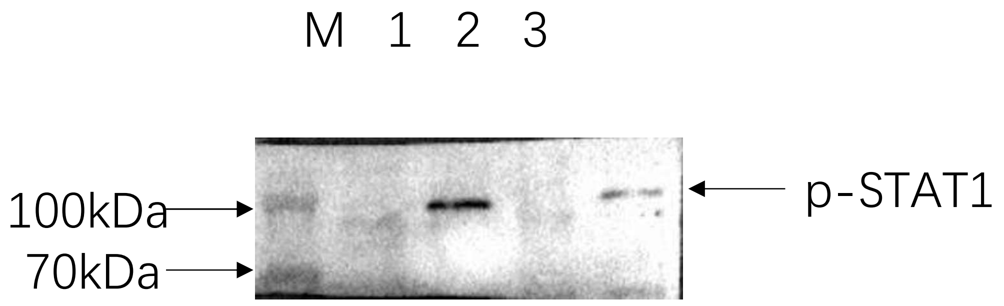

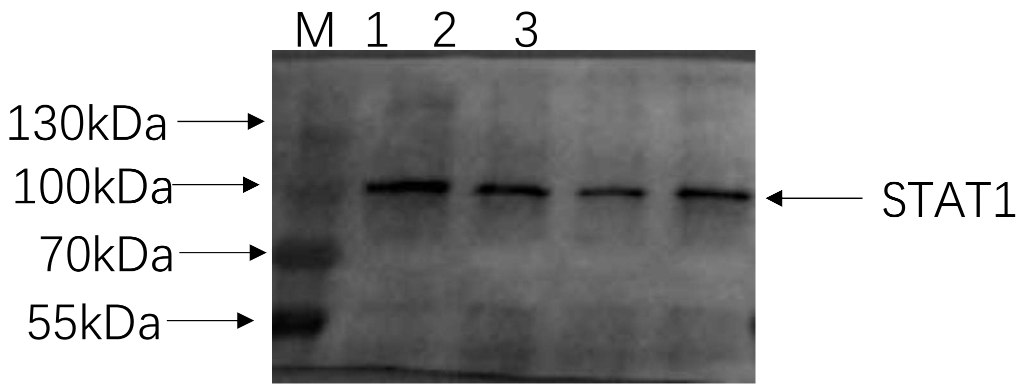

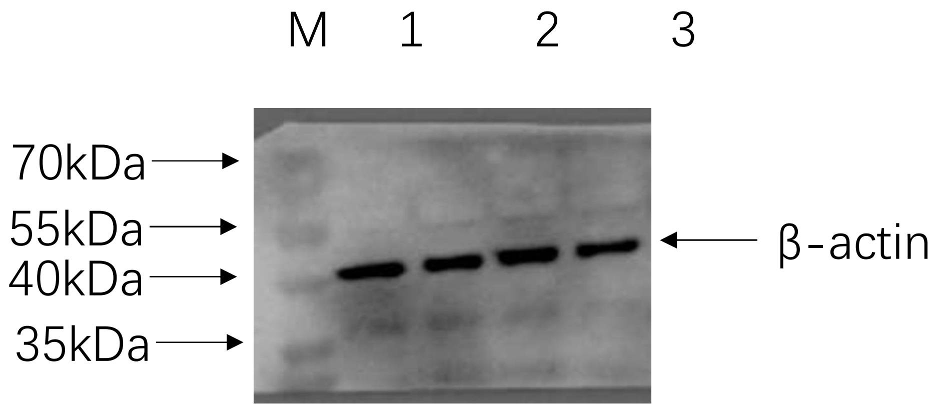

M: Prestained Protein Ladder

1： Uninfected control TM3 cells, 48 hpi

2: JEV-infected (MOI=1) TM3 cells, 48 hpi

3： Uninfected control CD4.KD cells, 48 hpi

4: JEV-infected (MOI=1) CD4.KD cells, 48 hpi
