# Supplementary material for: CD4 Molecule Plays an Important Role in the Inflammatory Response Induced by Japanese Encephalitis Virus Infection
Source: Vet Sci. 2026 Mar 9;13(3):254. doi: 10.3390/vetsci13030254 (PMC13030670; doi:10.3390/vetsci13030254)
Supplement: Supplementary file 1 [file vetsci-13-00254-s001.zip › vetsci-4166193-supplementary/figure S7.pdf]

FIGURE 7 A

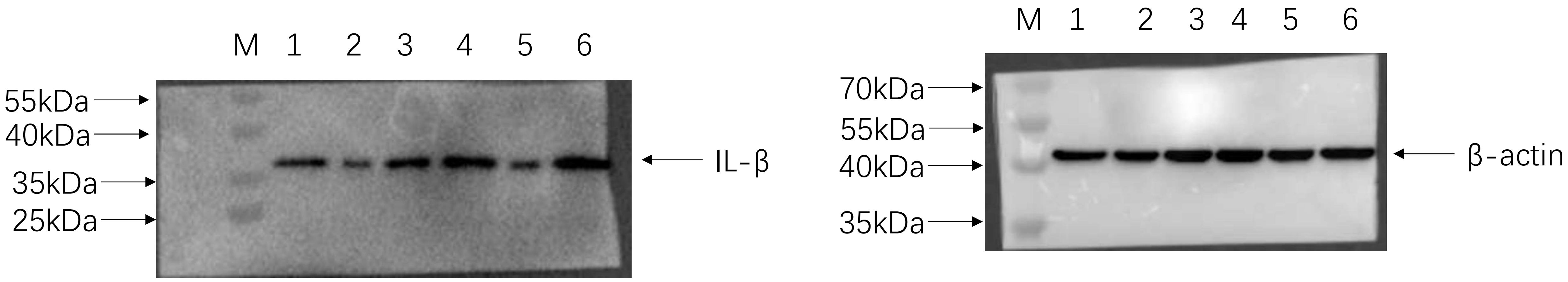

M: Prestained Protein Ladder
1：JEV-infected (MOI=1) TM3 cells, 48 hpi
2: JEV-infected (MOI=1) CD4.KD cells, 48 hpi
3：Pegfp-N1-CD4-transfected, JEV-infected (MOI=1) CD4.KD cells, 48 hpi
4：R08191-pretreated, JEV-infected (MOI=1) TM3 cells, 48 hpi
5：R08191-pretreated, JEV-infected (MOI=1) CD4.KD cells, 48 hpi
6：R08191-pretreated, Pegfp-N1-CD4-transfected, JEV-infected (MOI=1) CD4.KD cells, 48 hpi
