# Supplementary figures and images for: CD4 Molecule Plays an Important Role in the Inflammatory Response Induced by Japanese Encephalitis Virus Infection
Source: Vet Sci. 2026 Mar 9;13(3):254. doi: 10.3390/vetsci13030254 (PMC13030670; doi:10.3390/vetsci13030254)

FIGURE 1 C

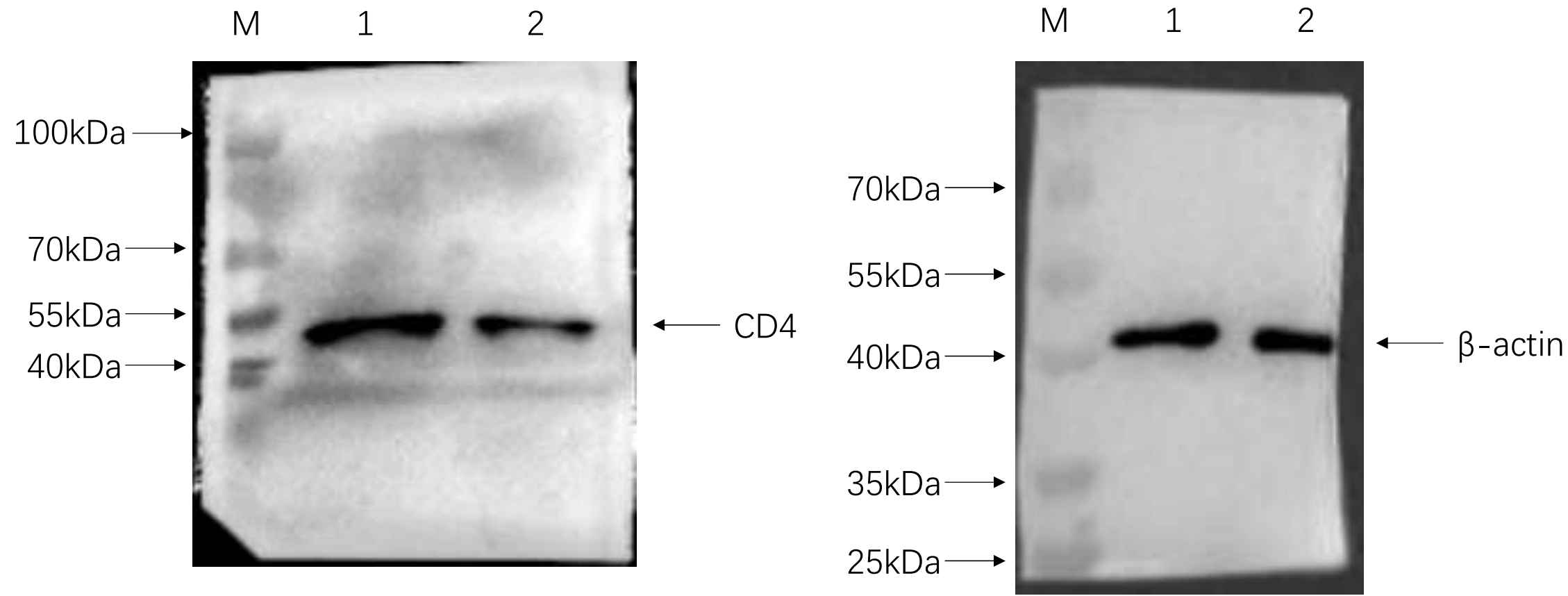

M: Prestained Protein Ladder  
1: TM3 cells  
2:CD4 knockdown cells

FIGURE 1 E

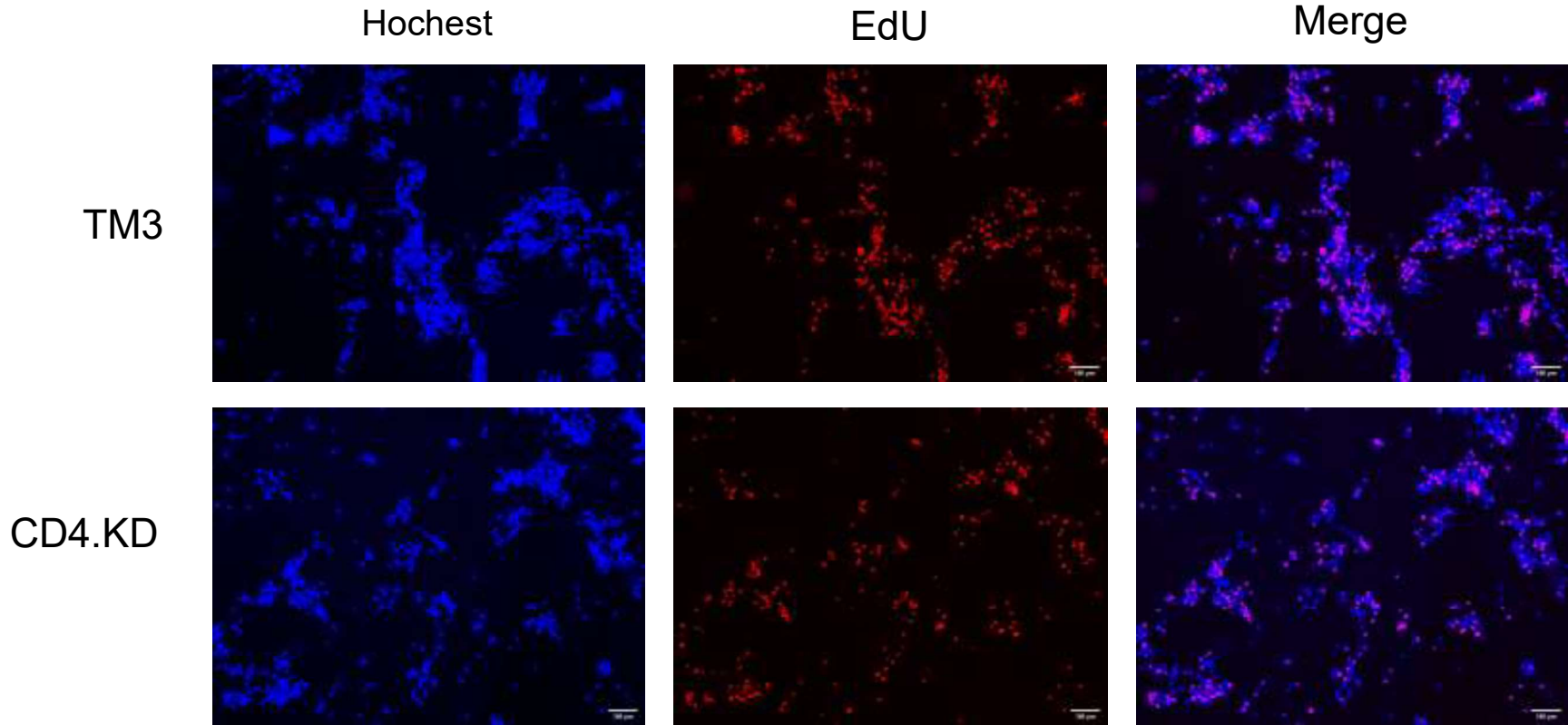

Supplement: Supplementary file 1 [file vetsci-13-00254-s001.zip › vetsci-4166193-supplementary/figure S1.pdf]

FIGURE 4 C

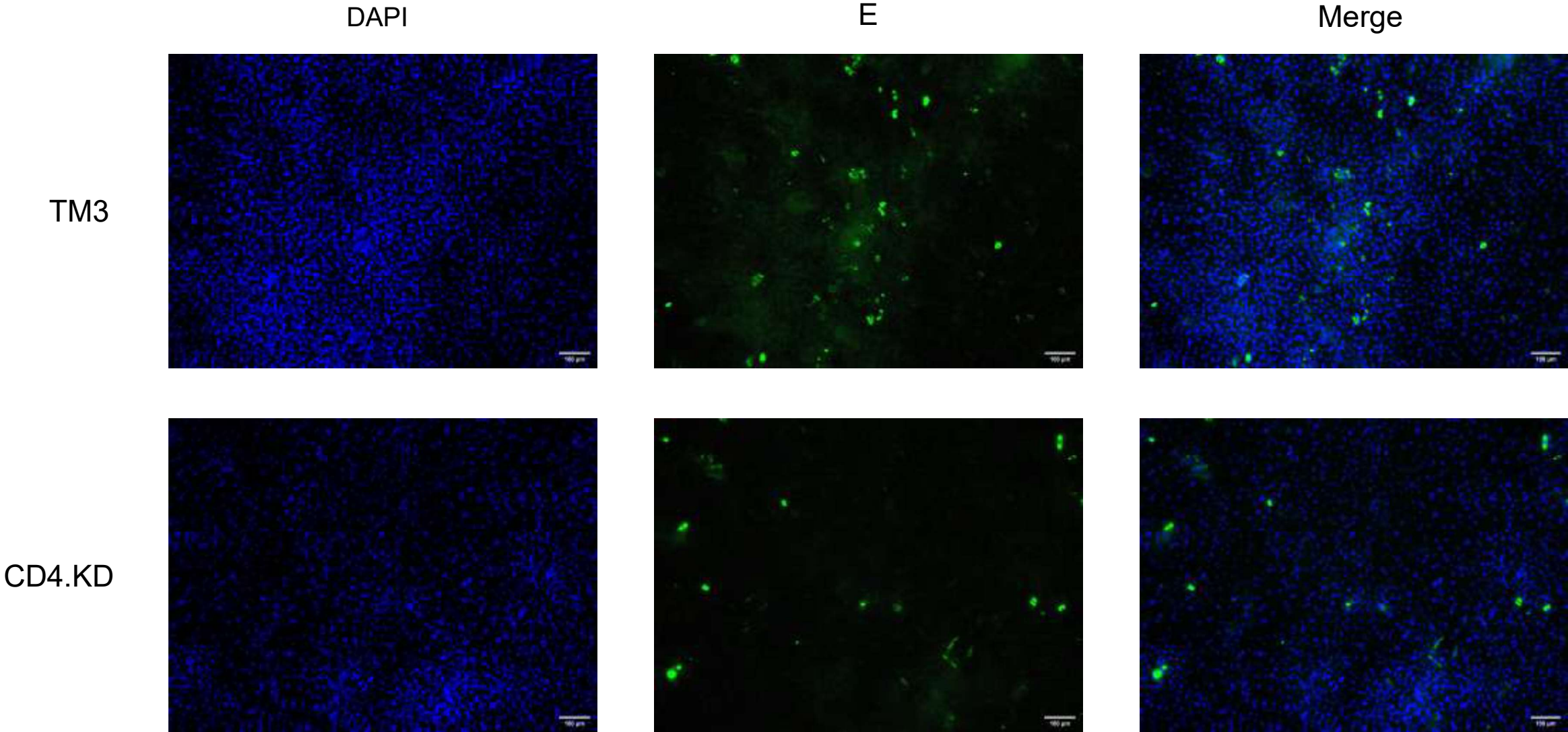

FIGURE 4 D

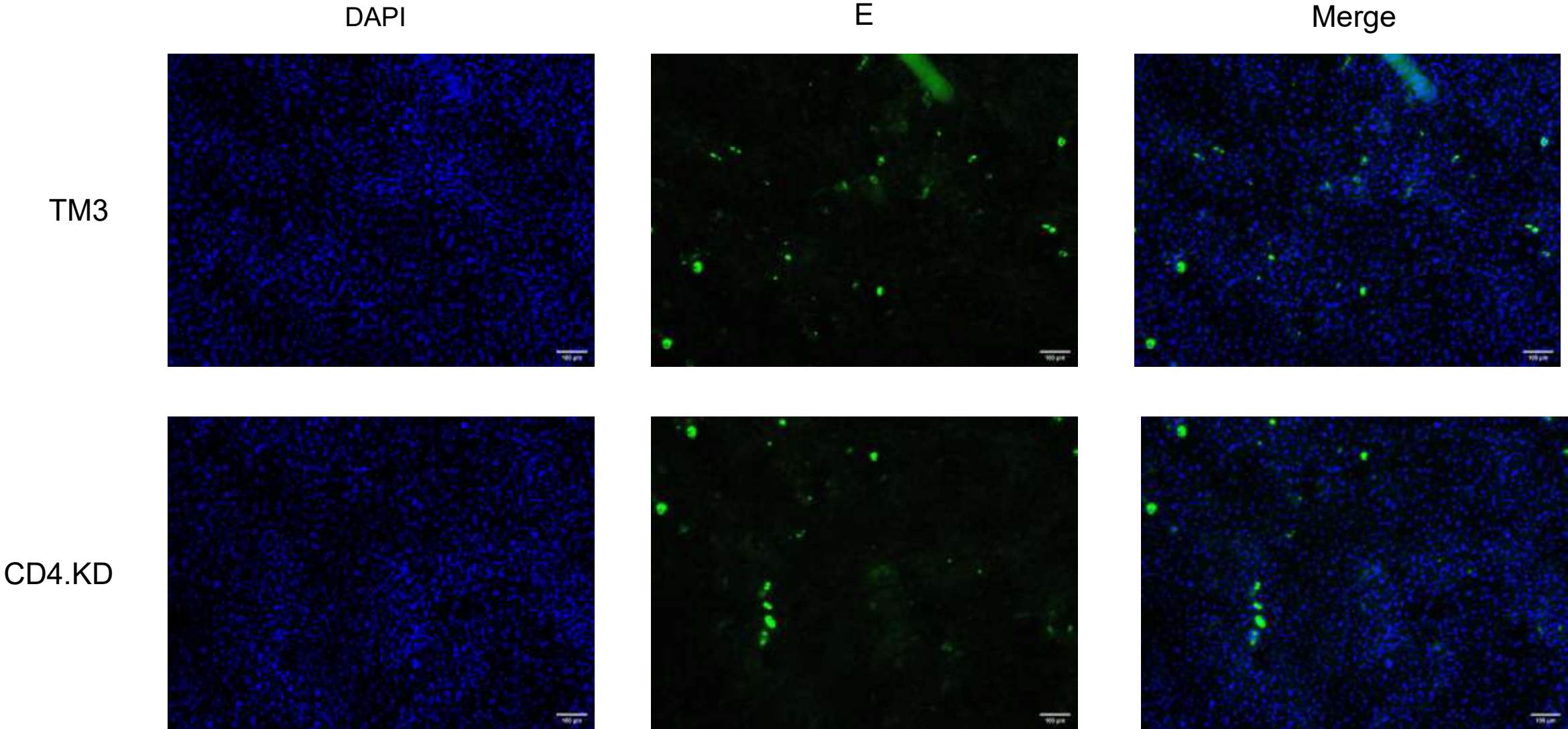

Supplement: Supplementary file 1 [file vetsci-13-00254-s001.zip › vetsci-4166193-supplementary/figure S4.pdf]

FIGURE 6 A

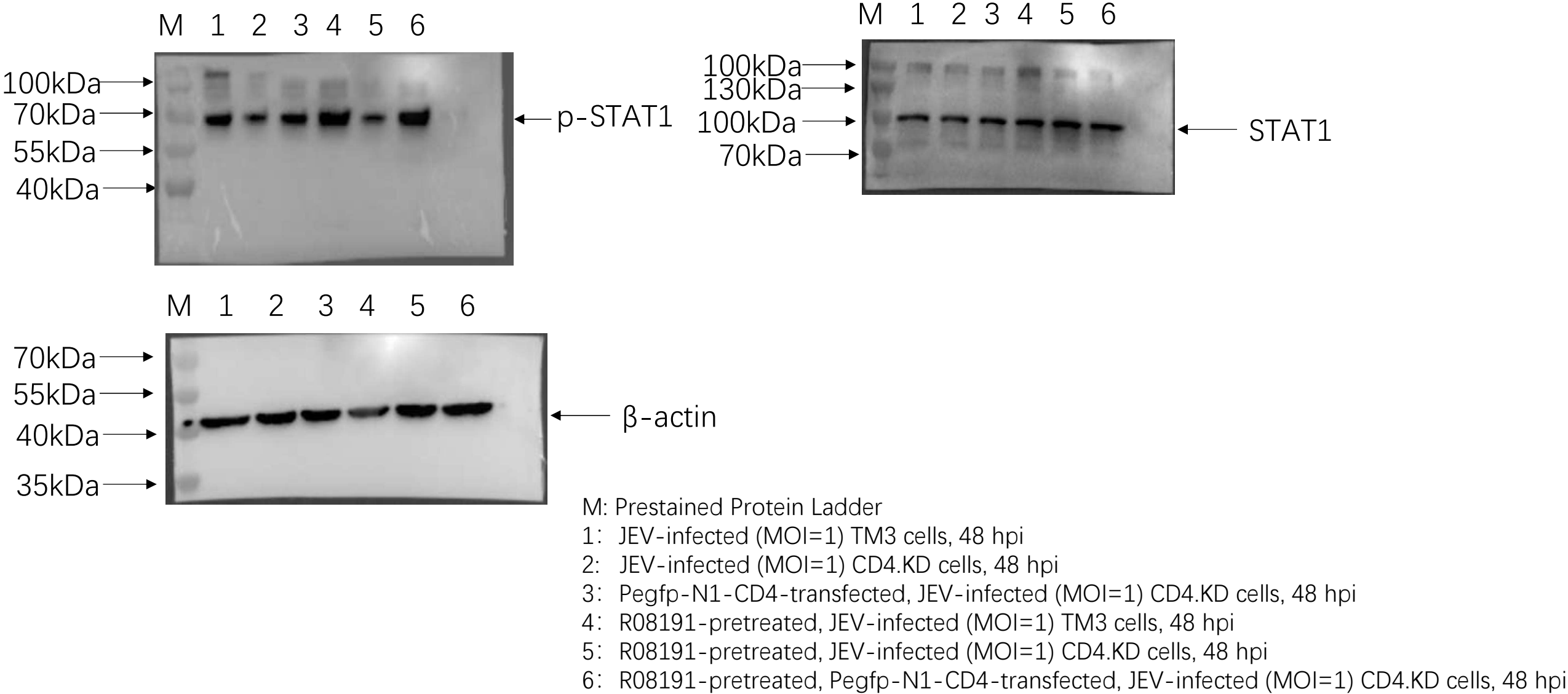

Supplement: Supplementary file 1 [file vetsci-13-00254-s001.zip › vetsci-4166193-supplementary/figure S6.pdf]
